# Indirect Tumor Inhibitory Effects of MicroRNA-124 through Targeting *EZH2* in The Multiple Myeloma Cell Line

**DOI:** 10.22074/cellj.2020.6492

**Published:** 2019-09-08

**Authors:** Javid Sabour Takanlu, Arad Aghaie Fard, Saeed Mohammdi, Seyed Mohammad Ali Hosseini Rad, Saeid Abroun, Mohsen Nikbakht

**Affiliations:** 1 Department of Hematology, Faculty of Medical Sciences, Tarbiat Modares University, Tehran, Iran; 2 Hematology, Oncology and Stem Cell Transplantation Research Center, Tehran University of Medical Sciences, Tehran, Iran; 3 Hematologic Malignancies Research Center, Tehran University of Medical Sciences, Tehran, Iran; 4 Department of Microbiology and Immunology, University of Otago, Dunedin, New Zealand

**Keywords:** Cyclin-Dependent Kinase Inhibitor 2A, Enhancer of Zeste Homolog 2, Multiple Myeloma, MicroRNA

## Abstract

**Objective:**

Multiple myeloma (MM) is an incurable plasma cell malignancy. Several genetic and epigenetic changes affect numerous critical genes expression status in this disorder. *CDKN2A* gene is expressed at low level in almost all cases with MM disease. The mechanism of this gene down-regulation has remained controversial. In the present study, we targeted *EZH2* by microRNA-124 (miR-124) in L-363 cells and assessed following possible impact on *CDKN2A* gene expression and phenotypic changes.

**Materials and Methods:**

In this experimental study, growth inhibitory effects of miR-124 were measured by MTT assay in L-363 cell line. Likewise, cell cycle assay was measured by flowcytometery. The expression levels of *EZH2* and *CDKN2A* were evaluated by real-time quantitative reverse-transcription polymerase chain reaction (qRT-PCR).

**Results:**

qRT-PCR results showed induction of *EZH2* gene expression after transduction of cells with lentivector expressing miR-124. The expression of *CDKN2A* was also upregulated as the result of EZH2 supression. Coincide with gene expression changes, cell cycle analysis by flow-cytometry indicated slightly increased G1-arrest in miR- transduced cells (P<0.05). MTT assay results also showed a significant decrease in viability and proliferation of miR- transduced cells (P<0.05).

**Conclusion:**

It seems that assembling of H3K27me3 mark mediated by EZH2 is one of the key mechanisms of suppressing CDKN2A gene expression in MM disease. However, this suppressive function is applied by a multi-factor mechanism. In other words, targeting EZH2, as the core functional subunit of PRC2 complex, can increase expression of the downstream suppressive genes. Consequently, by increasing expression of tumor suppressor genes, myeloma cells are stopped from aberrant expansions and they become susceptible to regulated cellular death.

## Introduction

Multiple myeloma (MM) is a malignant proliferation of monoclonal plasma cells characterized by clinical complications including hyper-calcaemia, renal dysfunction, anemia, and bone disease (CRAB) (1). MM is an intricate disease driven by the accumulation of several genetic and epigenetic changes. Whole oncogenomic studies of MM showed the presence of many highly recurrent and pivotal amplifications and/or deletions in genomic regions including the genes that are proposed to be involved in MM pathogenesis and progression (2). 

Aberrations in G1/S checkpoint of the cell cycle caused by either loss of tumor suppressor genes -such as retinoblastoma (Rb) and P16- or enhanced activity of cyclin D1 or CDK4/6 in the cell-cycle machinery, lead to neoplastic progression. One of the most frequently affected components of this pathway is P16. *CDKN2A* gene, encoding P16 tumor suppressor and located at 9p21, has been shown to be dysregulated in several neoplasias by deletions, point mutations and promoter hypermethylation (3, 4). Additionally, this tumor suppressor gene defective performance may be imperative for transformed phenotype commencement and maintenance in numerous neoplasms (5). Hence, it seems this gene has a crucial role in the initiation and progression of different malignancies, such as MM. 

In the recent years, there has been an increasing interest in epigenetic impacts on cancer which can be described as a disease with gene expression alterations. DNA methylation, histone modifications and noncoding RNAs are examples of epigenetic elements contributing to the pathobiology of MM through gene expression changes (6). 

Different DNA related procedures, such as transcription and replication, are affected by post-translational histone modifications (7). Several kinds of histone modifications -methylation, acetylation, phosphorylation, etc. based on the type and particularly affected residue, have a distinct influence on genes expression profile (8). In this study, we focused on a histone silencing mark -trimethylation of lysine on position 27 of histone 3 (H3K27me3)- which is mediated by polycomb repressive complex 2 (PRC2) catalytic subunit, EZH2 (9). 

Altered expression of EZH2 has been reported in various cancers. EZH2 overexpression frequently occurs in solid tumors whereas its down-regulation happens in hematological malignancies (10). Hence, depending on the type of malignancies and its role in cancer progression, EZH2 can be considered as onco/tumor suppressor gene. The mechanisms of these misregulations are different. For example in MM, interleukin-6 (IL-6) and c-Myc activation can mediated EZH2 up-regulation (11, 12). Different subsets of genes, having important roles in MM pathogenesis, are affected by EZH2 silencing impact.

microRNAs (miRNAs) are non-coding RNAs that have a crucial role in the regulation of gene expressions, particularly at the post-transcriptional level. These tiny gene regulators play an important role in carcinogenesis. Several studies have shown down-regulation of miR-124 in different types of cancers including hematological malignant disorders (13, 14). 

miR-124 was previously introduced as a direct repressor of *EZH2* and its expression is decreased in 50% of myeloma cell lines (14-16). This study aims to reveal the positive effect of miR-124 on *CDKN2A* gene expression through targeting *EZH2* gene and also evaluate phenotypic changes in myeloma cell line. 

## Materials and Methods

### Bacterial culture and plasmid extraction

E. Coli (DH5α) containing Lenti-miR-GFP-hasmiR- 124, pLenti-III-GFP-mir-control, psPAX2 and pMD2G plasmids (abm Inc., Canada) were cultured in LB-ampicillin broth and LB-kanamycin broth (Merck Darmstadt, Germany), respectively and incubated in shaker-incubator at 37°C at 120 rpm. After that, plasmid extraction was done using a DNA purification kit (NucleoBond^R^ Xtra Midi, MACHERY-NAGEL, Germany) according to the manufacturer’s instructions. 

### Transfection and virus packaging

**Fig.1:**
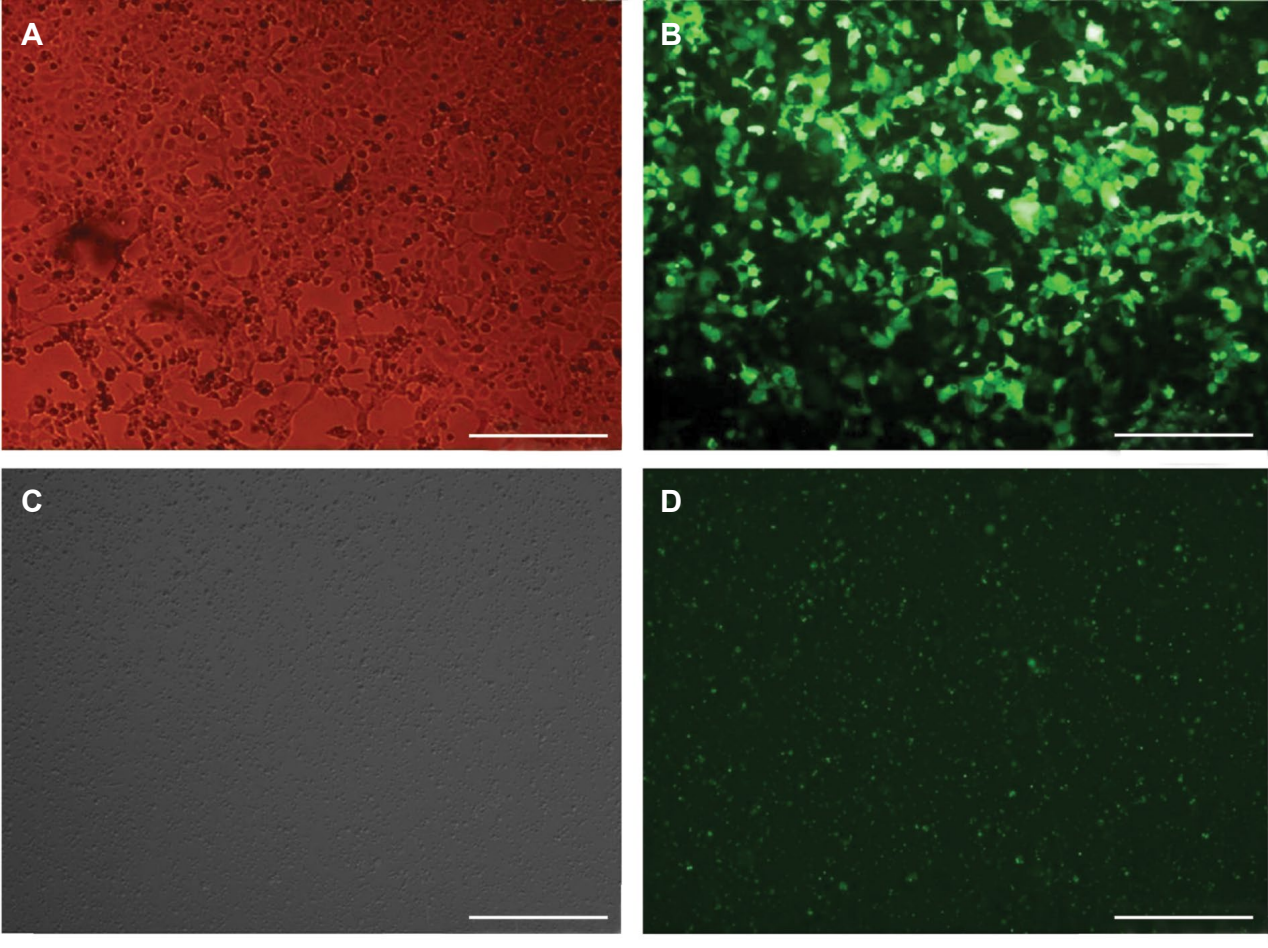
Light and fluorescent microscopic pictures of HEK293T and L-363 cells 48 hours post-transfection (×10). A. Light microscopic picture of the HEK cells (scale bar: 100 µm), B. The HEK cells transfected with pLenti-III-mir-GFP-124 (scale bar: 100 µm), C. Light microscopic picture of the L-363 cells, 48 hours post-transduction (×4), and D. The L-363 cells transduced with pLenti-III-miR-GFP-has-miR-124 (scale bar: 200 µm).

In this experimental study, for virus packaging, HEK293T cells were grown in DMEM cell culture media (Gibco, USA) supplemented with 10% fetal bovine serum (FBS), 100 units/ml penicillin (Pen), 100 mg/ml streptomycin (Strep, all from Gibco, USA) and incubated in 37°C with 5% CO_2_. To passage, HEK293T cells were separated from flask by Trypsin-EDTA (Gibco, USA) and after two passages, HEK293T cells with confluency of about 70-80% were used for virus packaging. PsPAX2 plasmid comprising of the gag/pol packaging genes and pMD2.G plasmid composed of VSV-G were co-transfected with pLenti-III-miR-GFP-has-miR-124 (also pLenti-IIIGFP- mir-control vector) by calcium phosphate transfection method, as previously described (Fig .1A, B) (17). Viral supernatant was collected every 12 hours post-transfection until 72 hours, and it also was centrifuged (3000×g for 10 minutes at 4°C) to remove cell debris. Finally, viruses were concentrated using ultracentrifugation at 21000 rpm at 4°C for 3 hours. Viral titration was performed on HEK293T cells with a serial dilution of the viral stock. Virus stock was aliquoted and it was frozen at -70°C for further use. 

### Cell culture and transduction

HEK293T and L-363 myeloma cell lines were purchased from Pasture Institute (Iran). L-363 cells were cultured in RPMI-1640 (Gibco, USA) supplemented with 10% FBS and 1% Pen/Strep. They were then cultured at 37°C in a 5% CO_2_ incubator. For stable expression of miR-124, L-363 cells were transducted with lentiviruses by spinoculation protocol which increases transduction efficiency in the presence of 6 mg/ml polybrene (Sigma-Aldrich, USA) (Fig .1C, D). 

### RNA extraction, cDNA synthesis and quantitative reverse-transcription polymerase chain reaction

Total RNA was extracted using QIAzol Reagent (Qiagen, USA) according to the manufacturer’s instructions. Total RNA-including miRNAs and mRNAs were used for cDNA synthesis following the manufacturer’s protocol (Thermo Scientific, USA). For miRNA, reverse transcription was performed using a miRNA 1^st^-Strand cDNA Synthesis Kit (Stratagene, Agilent Technologies Inc., USA). The cDNA samples were subjected to quantitative reverse-transcription polymerase chain reaction (qRT-PCR, EvaGreen-based qRT-PCR, USA) using High-Specificity miRNA qRT-PCR Detection Kit (Stratagene, Agilent Technologies Inc., USA). Relative expression levels of miRNAs were normalized to SNORD-47 as an endogenous control. For mRNAs, *ß-ACTIN* was used as reference gene in the qRT-PCR reaction. In the next step, PCR and qRTPCR were done in order to evaluate miR-124, *EZH2* and *CDCN2A* expression levels. Taq DNA polymerase 2x Master Mix Red and Real Q-PCR 2x Master Mix Kit (Amplicon, Denmark) was used for PCR and qRT-PCR, respectively. Relative expression levels of miRNA and other genes were calculated using the 2^-ΔΔCt^ method. The primer sequences are provided in Table 1. 

### Flow cytometric analysis

**Fig.2:**
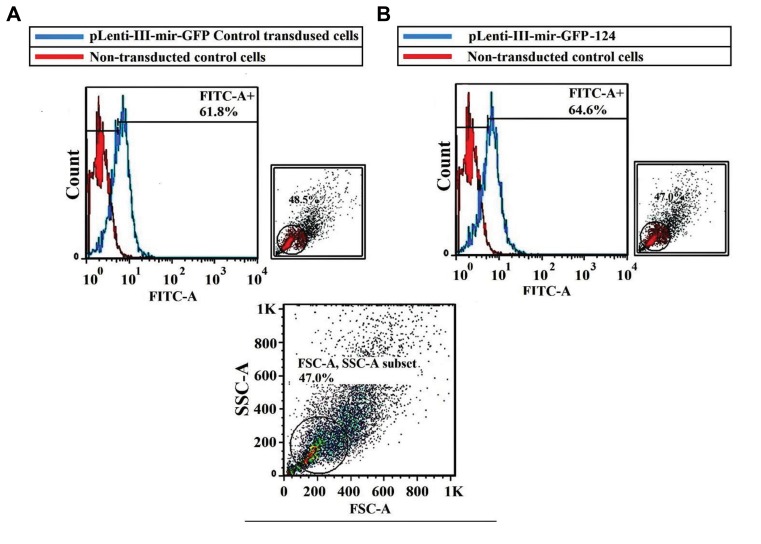
Flow cytometric data of virus transduced L-363 cells 48 hours post-transduction. A. PLenti-III-GFP-mir-control transduced cells and B. PLenti-III-miR- GFP-has-miR-124 transduced cells. Results show 61.8% and 64.6% GFP-expressing cells for control and miR-transduced cells, respectively.

Flow cytometry was used for both evaluations of GFP- expressing transduced cells and cell cycle analysis.48 hours post-transduction, L-363 cells were checked to find GFP-positive cells (Fig .2). In order to analyze cell cycle, the L-363 cells were fixed with cold (20°C) 70% ethanol. Afterward, the cells were washed in phosphate buffered saline with tween-20 (PBST). They were suspended again in 0.5 ml PBST, comprising 20 µg/ml RNase, and incubated at 37°C for 40 minutes. Then, the cells were stained with 20 µg/ml propidium iodide (PI) for 30 minutes at 37°C. DNA quantity was measured using a Flow-cytometry (BD Biosciences, USA). 

**Table 1:** List of the primers used in quantitative reverse-transcription polymerase chain reaction (qRT-PCR) analyses


Genes	Primer type	Primer sequence (5ˊ-3ˊ)

*hsa-miR124-3p*	stem-loop RT primer (For cDNA synthesis)	GTC GTA TCG AGA GCA GGG TCC GAG GTA TTC GCA CTC GAT ACG ACG GCA TT
	Forward	GCT AAG GCA AGC GGT G
	Reverse (Common for both miR and Snord)	GAG CAG GGT CCG AGG T
*SNORD-47*	RT	GTC GTA TGC AGA GCA GGG TCC GAG GTA TTC GCA CTG CAT ACG ACA ACC TC
	Forward	ATC ACT GTA AAA CCG TTC CA
*EZH2*	Forward	TAC TTG TGG AGC CGC TGA C
	Reverse	CTG CCA CGT CAG ATG GTG
*CDKN2A*	Forward	CCC AAC GCA CCG AAT AGT TA
	Reverse	ACC AGC GTG TCC AGG AAG
*B-ACTIN*	Forward	CTG GAA CGG TGA AGG TGA CA
	Reverse	AAG GGA CTT CCT GTA ACA ATG CA


### Proliferation assay

MTT assay was done in 96-well plates for evaluating cellproliferation. Briefly, the L-363 cells (5×10^3^ per well) wereseeded in 100 µl culture medium in a 96-well plate. Then, 10µl MTT (5 mg/ml in PBS, Sigma-Aldrich, USA) was addedto each well and incubated at 37°C for 3 hours. At the end, the supernatant was changed with 100 µl dimethyl sulfoxide(DMSO, Sigma-Aldrich, USA) and absorption of viable cellswere measured at 570 nm using a microplate ELISA reader(Labomed, USA). The wells containing only DMSO (without cells) have been used as a blank.

### Statistical analysis

The obtained data were analyzed by SPSS 18.0. Student’s t test was used to compare the results. All data are presented as means ± standard error (SE) of triplicate determinant. P<0.05 was considered statistically significant in all experiments.

## Results

### Expression level of miR-124 in L-363 cells after transduction 

**Fig.3:**
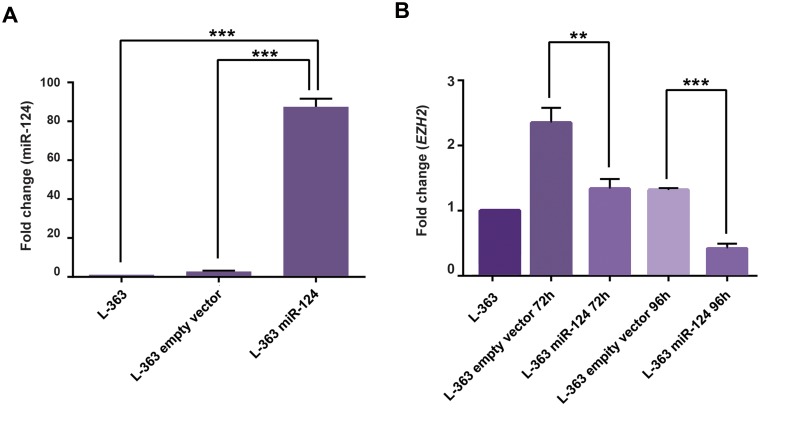
miR-124 and *EZH2* expression fold changes before and after transduction. A. Expression levels of miR-124 evaluated by quantitative reverse- transcription polymerase chain reaction (qRT-PCR) in the transduced L-363 cells with miR-124 in comparison with the empty vector group and the cells without transduction (after 72 hours) and B. Expression levels of EZH2 evaluated by qRT-PCR in L-363 cells transduced with miR-124 in comparison with the empty vector transduced group and the cells without transduction (after 72 and 96 hours). **; P<0.01 and ***; P<0.001. Experiments were performed at least three times, independently.

To evaluate up-regulation of miR-124 after transduction, expression level of this microRNAwas assessed by qRT-PCR in transduced (as well as non-transduced) L-363 cell line after 72 hours post-transduction. Comparing the results of pLentiIII- miR-GFP-has-miR-124 and pLenti-III-GFP-mir-control vector-transduced cells with non-transduced L-363 cells, relatively showed respectively about 2.8 ± 0.2 and 87.4 ± 2.4 fold expression changes (Fig .3A). 

### Gene expression analyses of *EZH2* and *CDKN2A*

*EZH2* and *CDKN2A* gene expressions were evaluated after 72 hours and 96 hours post-transduction. *EZH2* gene expression in pLenti-III-mir-GFPcontrol vector and pLenti-III-miR-GFP-has-miR-124 transduced cells showed about 2.3 ± 0.13 and 1.3 ± 0.08 fold after 72 hours (P<0.01) and also about 0.4 ± 0.04 and 1.3 ± 0.01 fold after 96 hours (P<0.001), respectively (Fig .3B). These changes showed down-regulated status of *EZH2* in the miR-transduced cells compared to the control group. Fold changes were calculated in comparison with the non-transduced cells. qRT-PCR analysis of *CDKN2A* gene showed interesting results, including no detectable expression in none of the non-transduced and transduced cells, except the cells evaluated 96 hours after transduction. The expression level of *CDKN2A* was changed with a delay. Thus, 96 hours after forced expression of miR-124, *CDKN2A* level showed up-regulation.

### miR124- overexpression effect on cell cycle

**Fig.4:**
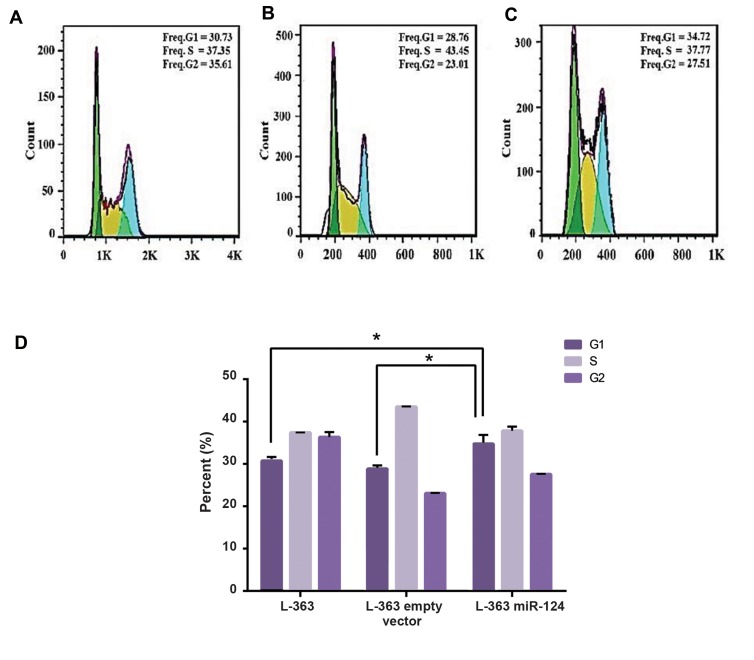
Cell cycle analysis of L-363 cells before and after transduction of miR-124. A. L-363 cells before transduction, B. Empty vector-transduced L-363 cells, C. miR-124 transduced-L-363 cells (means ±SE), and D. Representative bar graph of the L-363 cells cycle before and after transduction. *; P<0.05.

Similar to the gene expression analysis, three groupswere studied for cell cycle analysis. Flow-cytometric datashowed perturbations in pLenti-III-miR-GFP-has-miR-124transduced cells in comparison with pLenti-III-GFP-mircontrol vector and non-transduced cells. It seems that miR124 overexpression increases the percentage of cells inG1 phase with a concomitant reduction in the percentageof cells in the S phase. About 34.72% ± 1.2% of L-363cells expressing miR-124 were arrested in G1 phase, incomparison with 28.76% ± 0.5% and 30.73% ± 0.54% forpLenti-III-GFP-mir-control vector and non-transduced cells, respectively (P<0.05, Fig .4). 

### Cellular viability and proliferation rate change after miR-124 induction

**Fig.5:**
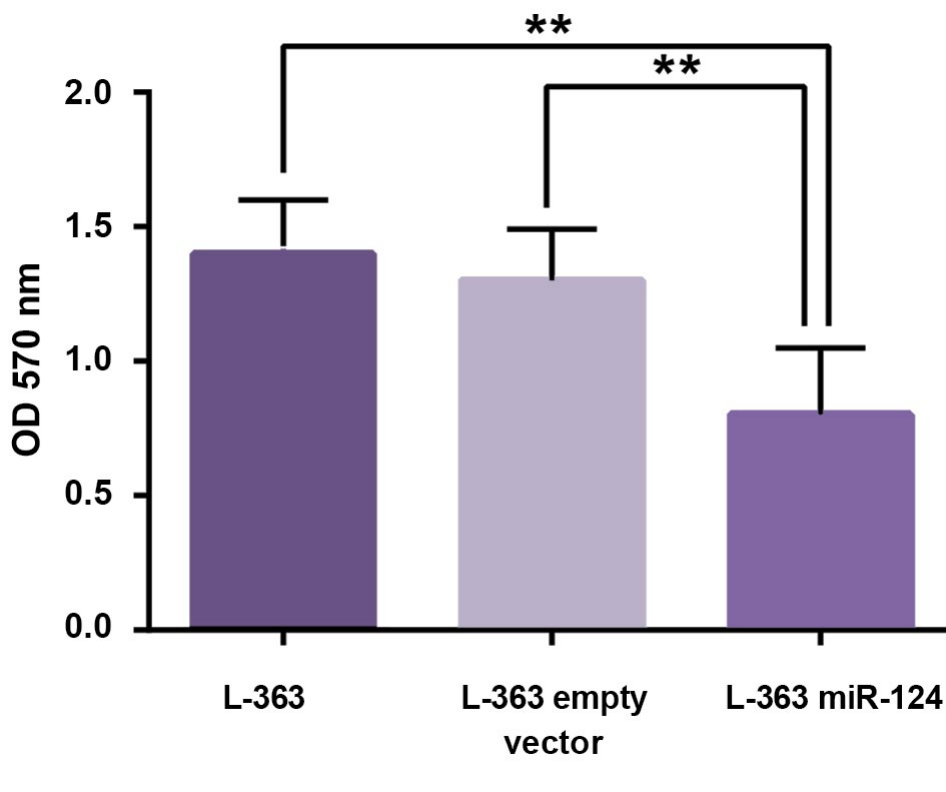
MTT assay results in 96 hours post-transduction. L-363 cells transduced with miR-124 show decreased absorbance in 570 nm compared to the control groups. **; P<0.01.

We used MTT assay to estimate cell viability and proliferation. In line with cell cycle results, MTT assay showed a significant decline in the viability and proliferation of cells with an elevation of miR-124 level (Fig .5).

## Discussion

Accumulating data showed changing epigenome in malignancies. So this area of research could be considered as a promising approach for the treatment of various cancers (18, 19). Without direct changing of DNA sequences, epigenetic mechanisms have the robust capability to control different genes expression status. In this study, we attempted to reveal another role of miR-124 in epigenetic of MM disease, through modulation of core enzymatic subunit of PRC2, EZH2, in gene expression status of *CDKN2A* locus. Previously Zhan et al. (20) showed that EZH2 level along with 30 other genes has different expression status in normal and malignant plasma cells. Moreover, a straight correlation between EZH2 level with cancer progression has been detected (9). 

miR-124 was recognized to be down-regulated in many types of cancer. It has also been reported as tumor suppressor microRNA. It has been shown that the miR124 expression is decreased in leukemic cell lines e.g. MM cell (14). Among 9217 target genes predicted for miR-124 in microRNA.org (http://www.microrna.org), 3'-UTR of EZH2 has been shown to have a complementary sequence for binding to miR-124. In line with the previous studies, our results confirmed that *EZH2* is a target gene of miR124 (15, 16).

Numerous studies have reported down-regulation of *CDKN2A* in almost all MM cases, despite infrequent genetic aberrations of the related gene. Promoter hypermethylation of the *CDKN2A* occurs only in 40% of patient with MM (21-23). Additionally, promoter methylation of *CDKN2A* does not seems to be the sole or at least the main element of silencing this locus, since even the cases without promoter methylation status express low level of *CDKN2A* gene (3, 22). Through specific inhibiting EZH2 expression, mediated by miR-124, we suggested that this histone modifying enzyme can be among the key elements causing *CDKN2A* low expression in MM. We showed that inhibition of PRC2 complex through targeting *EZH2* by miR-124 would lead to increased expression level of *CDKN2A* gene. This result was consistent to Overhoff et al. (24) who found a positive feedback loop between senescence-associated miRNAs targeting *EZH2* and inducing *CDKN2A* gene in both human epithelial cells and fibroblasts. These findings suggest a potential approach for recovery of *CDKN2A* expression level by targeting epigenetic suppressor complexes in MM disease. 

There are several cell cycle studies showing that G1 controlling proteins which are suppressed in most MM cases. This suggests the critical role of negative cell cycle checkpoint regulators, such as P16 in MM’s pathogenesis (3, 21, 25). By inhibiting EZH2, through overexpression of a miRNA, we proposed a simple and efficient strategy to increase *CDKN2A* expression. This resulted in a decrease of proliferation and viability of myeloma cell line. We showed that indirect up-regulation of *CDKN2A* gene, through exogenous expression of miR-124, resulted in increasing the number of cells accumulated in the G1 phase of the cell cycle. Moreover, it was shown that prolonged G1 arrest would diminish anti-apoptotic proteins like IRF4, which protecting myeloma cells from apoptosis or decreasing chemo-resistance (26). 

It has been previously determined that *INK4b-ARF- CDKN2A* locus encoding three important tumor suppressors, P15^INK4b^, P14^ARF^, and P16^INK4a^ is tightly controlled (27). Different factors participate in the regulation of this locus along with PRCs (PRC1 and PRC2), including long non-coding RNAs (lncRNAs), specially ANRIL (28). So, for sufficient elevation of gene expression levels in this locus, targeting two or more molecules could likely reinforce arbitrary impacts on *CDKN2A*. 

## Conclusion

Collectively, *CDKN2A* is a vital controller of the cell cycle in malignant plasma cells. It is negatively affected by suppressive histone marks, through PRC complexes. miR-124 is able to eliminate adverse impacts on the expression level of *INK4b-ARF-CDKN2A* locus through targeting EZH2. However, multiple factors are involved in PRC2-mediated histone changes; therefore, other factors like structural subunits of PRC complex as well as ANRIL, working as an scaffold for PRC complexes, can be targeted along with EZH2. It can also be recommended for future researches that miR-targeted cells can be treated with chemotherapeutical agents coincidently and following analysis can reveal efficiency of this anti cancer strategy. 
